# Production, Characterization and Application of Oxide Nanotubes on Ti–6Al–7Nb Alloy as a Potential Drug Carrier

**DOI:** 10.3390/ma14206142

**Published:** 2021-10-16

**Authors:** Bożena Łosiewicz, Agnieszka Stróż, Patrycja Osak, Joanna Maszybrocka, Anna Gerle, Karolina Dudek, Katarzyna Balin, Dariusz Łukowiec, Maciej Gawlikowski, Sylwia Bogunia

**Affiliations:** 1 Institute of Materials Engineering, Faculty of Science and Technology, University of Silesia in Katowice, 75 Pułku Piechoty 1A, 41-500 Chorzów, Poland; agnieszka.stroz@us.edu.pl (A.S.); patrycja.osak@us.edu.pl (P.O.); joanna.maszybrocka@us.edu.pl (J.M.); 2 Łukasiewicz Research Network, Institute of Ceramics and Building Materials, Refractory Materials Division, Toszecka 99, 44-100 Gliwice, Poland; a.gerle@icimb.pl (A.G.); k.dudek@icimb.pl (K.D.); 3 The August Chełkowski Institute of Physics, University of Silesia in Katowice, 75 Pułku Piechoty 1A, 41-500 Chorzów, Poland; katarzyna.balin@us.edu.pl; 4 Faculty of Mechanical Engineering, Silesian University of Technology, Konarskiego 18a, 44-100 Gliwice, Poland; dariusz.lukowiec@polsl.pl; 5 Foundation of Cardiac Surgery Development, Artificial Heart Laboratory, Wolności 345a, 41-800 Zabrze, Poland; mgawlik@frk.pl; 6 Old Machar Medical Practice, 526-528 King Street, Aberdeen AB24 5RS, UK; sylwia.bogunia@nhs.scot

**Keywords:** anodic oxidation, biomaterials, drug delivery system, gentamicin, oxide nanotubes, titanium alloys

## Abstract

This work concerns the development of a method of functionalization of the surface of the biomedical Ti–6Al–7Nb alloy by producing oxide nanotubes (ONTs) with drug-eluting properties. Shaping of the morphology, microstructure, and thickness of the oxide layer was carried out by anodization in an aqueous solution of 1 M ethylene glycol with the addition of 0.2 M NH_4_F in the voltage range 5–100 V for 15–60 min at room temperature. The characterization of the physicochemical properties of the obtained ONTs was performed using SEM, XPS, and EDAX methods. ONTs have been shown to be composed mainly of TiO_2_, Al_2_O_3_, and Nb_2_O_5_. Single-walled ONTs with the largest specific surface area of 600 cm^2^ cm^−2^ can be obtained by anodization at 50 V for 60 min. The mechanism of ONT formation on the Ti–6Al–7Nb alloy was studied in detail. Gentamicin sulfate loaded into ONTs was studied using FTIR, TG, DTA, and DTG methods. Drug release kinetics was determined by UV–Vis spectrophotometry. The obtained ONTs can be proposed for use in modern implantology as carriers for drugs delivered locally in inflammatory conditions.

## 1. Introduction

The progress of civilization has led to increased requirements for medical care, which should become more effective in solving the many health problems of an aging society. One of the answers to the challenges of modern medicine is the development of new biomaterials with increased corrosion resistance and biocompatibility, intended for long-term implants in implantology [1,2,3]. The most promising group of biomaterials for such applications is titanium and its single-phase alloys α or β and two-phase alloys α + β, which contain the additions of Al, V, Nb, Ta, Zr, Mo, Si, Sn, Pd, Fe, and Hf and exhibit osseointegrative properties [1,2,3,4,5,6,7,8,9,10,11]. The two-phase Ti–6Al–4V alloy has so far dominated the long-term implant market. However, the use of V as an alloy additive poses a threat to patients’ health due to the harmful effects of vanadium ions on the human body [4]. Vanadium is an element that, in trace amounts, is present in the human body as a microelement important in proper functioning. However, too high a concentration of V in the body can cause allergic reactions, kidney damage, and irritation of the digestive and respiratory systems. An excess of V can be toxic to the nervous system and brain cells and even cause manic-depressive psychosis. Vanadium can also inhibit many enzyme systems and exert genotoxic effects, adversely affecting various stages of fetal reproduction and development. 

Among vanadium-free titanium alloys, the Ti–6Al–7Nb alloy is of particular interest due to better toleration in the tissue environment [12,13,14,15,16,17,18,19,20,21]. The biotolerance of this alloy results from the properties of the passive layer composed of the most stable forms of Al (Al_2_O_3_) and Nb (Nb_2_O_5_) oxides. This two-phase structure (α + β) alloy is characterized by favorable mechanical properties, high corrosion resistance, and high biotolerance, which is extremely important in the case of titanium alloys susceptible to abrasive wear in the operation of cooperating elements in stressed joints. The Ti–6Al–7Nb alloy has been used in dentistry for the production of crowns and bridges. Elements made of this alloy exhibit similar tensile strengths as those made of Ti–6Al–4V alloy, while they have better corrosion resistance and are characterized by greater elongation. They also have lower porosity and greater hardness and wear resistance than pure titanium. The Ti–6Al–7Nb alloy is used in orthopedics for the production of heads and stems of joint endoprostheses. It is also used to make bone stabilizers, screws, and bone nails.

The condition of long-term success in implantological treatment is primarily osseointegration [22]. Bone integration of titanium implants is possible because the oxygen contained in the bone tissue forms a layer of biocompatible TiO_2_ on the surface of the titanium implant, on which new, mineralizing bone tissue can be deposited, forming the proper fixation of the implant. The introduction of the intraosseous implant into the bone causes traumatization of the bone tissue, which requires subsequent regeneration. Recent research in the field of implantology also shows that stabilization of the collagen scaffold, facilitating the build-up of bone tissue on the implant surface, occurs more easily on the rough surface of the endosseous implant. The area of mutual bone adhesion to the porous surface of the implant is also increased. This is why almost all new generation implants have a porous surface, obtained with the use of various technologies [1,2,3,5,6,7,8,9,10,12,13,14,15,16,17,18,19,20,21,22].

Local inflammation of the tissues around the implant occurs after the implantation procedure. In implant treatment, it is desirable that the drug reaches the site of action at a certain concentration and that the therapeutic dose remains constant for a sufficiently long time to achieve the desired effect. Standard methods of drug delivery do not fully exploit the therapeutic potential of drug substances. This is because the drug is distributed throughout the body when the drug is given, usually by the oral route. This reduces the chance of a large amount of the dose reaching its destination, which forces it to be increased in the applied preparation. For these reasons, new solutions are sought, characterized by an increased pharmacological response. One such solution is the use of intelligent carrier systems based on oxide nanotubes (ONTs), which act as drug-filled micro- or nanosyringes. This solution can have a huge impact on the effectiveness of implant treatment [23,24]. ONTs possess drug-eluting properties and can be proposed as prospective biomaterials for bone fixation and the reduction in bone infection [24].

Self-organized ONT layers on titanium and its alloys are produced by anodization, usually at a constant voltage in the range of 1–30 V in aqueous electrolytes or 5–150 V in non-aqueous electrolytes with the addition of fluoride ions (0.1–1 wt%) [6,7,8,9,10,12,13,14,15,16,18,19,20,21,23,24,25]. During this electrochemical oxidation process carried out in electrolytes containing fluoride ions on the anode surface, the oxidation and dissolution of metal oxides take place. The choice of electrolyte in which the anodic oxidation is conducted has the greatest impact on the microstructure and properties of the obtained ONTs.

The first-generation ONTs can be obtained from aqueous solutions that contain HF or its salt or mixtures of hydrofluoric acid with other acids, e.g., HNO_2_/HF, HNO_3_/HF, H_2_SO_4_/HF, H_2_Cr_2_O_7_/HF, H_3_PO_4_/HF. The first-generation nanotubular oxide structures can also be obtained from an electrolyte based on CH₃COOH with the addition of H_2_O and NH_4_F. The structures obtained by using this type of electrolyte are distinguished by low surface ordering and a limited thickness of 500–600 nm [9,12,16,23,25].

The high rate of chemical dissolution of the oxide layers in acid solutions leads to the limited growth of ONTs. For this reason, for the preparation of the second-generation ONTs, it was proposed to use buffer solutions with variable pHs, containing in their compositions fluorine salts in the form of NaF, KF, or NH_4_F instead of HF. The most commonly used electrolyte for the preparation of the second-generation ONTs is a mixture of Na_2_SO_4_ with the addition of NaF or (NH_4_)H_2_PO_4_ with the addition of NH_4_F. The pH of the electrolyte affects the rate of electrochemical etching and chemical dissolution. The optimal pH value of such solutions is 3–5. At pH values above 5, during the anodization of titanium and its alloys, the increased hydrolysis of titanium ions and deposition of a mixture of titanium hydroxides on the surface of ONT layers takes place, which is difficult to remove. The increase in pH increases the length of the obtained ONTs [6,7,8,9,14,21,23,25].

The compositions of electrolytes for the preparation of the third-generation ONTs are based on organic polar solvents such as formamide, N-methylformamide, ethylene glycol (EG), diethylene glycol, dimethylsulfoxide, methanol and glycerol with the addition of a source of fluorine ions in the form of HF, NH_4_F or quaternary ammonium salts of fluorine and H_2_O in 1–5%. The water content of these electrolytes is responsible for the dissolution rate of the top surface of ONTs. Reducing the amount of water contributes to the increase in the length of ONTs even up to 1000 μm. For some electrolytes containing glycerol or methanol, larger amounts of H_2_O (25–50%) are used. The use of electrolytes containing EG leads to the formation of ONTs with very smooth walls, while the use of glycerin solutions with various amounts of H_2_O allows the morphology and properties of the obtained ONTs to be controlled by selecting the viscosity [9,10,13,15,18,19,20,23,24,25]. 

The fourth-generation ONTs are produced from electrolytes that do not contain fluoride ions, mainly from an aqueous HCl solution or a mixture of HCl/H_2_O_2_. The length of the obtained ONTs is usually several hundred nanometers. Titanium anodization was also performed using electrolytes, a mixture of NH_4_Cl and HCl, H_2_SO_4_, C_2_H_2_O_4_, CH_2_O_2_, CCl_3_COOH, or C_6_H_12_O_7_. The length of the produced thin ONTs with poor adhesion to the substrate reached 60 μm, similar to that when using the electrolyte containing HClO_4_ [9,23,25]. The fourth-generation ONTs are obtained from the most eco-friendly electrolytes. However, the literature data show that bundles of thin ONTs on titanium with a diameter of only 15–35 nm were obtained in a narrow voltage range. Therefore, the most promising drug carriers among all generations of ONTs are the third-generation ONTs due to the most tunable microstructure and good adhesion to the substrate.

The present work considers the functionalization of the Ti–6Al–7Nb alloy by producing the third-generation ONTs on its surface to develop innovative long-term implants with extended drug-eluting ability. The anodizing parameters of the Ti–6Al–7Nb alloy in an electrolyte with a new chemical composition of EG, water, and ammonium fluoride, were optimized. The assessment of the obtained ONTs for use as gentamicin sulfate (GS) carriers for local antibiotic therapy in bacterial infection after implantation was carried out.

## 2. Materials and Methods

### 2.1. Material Preparation

The material under study was the commercial Ti–6Al–7Nb alloy (BIMOTECH, Wrocław, Poland) in the form of a bar with a diameter of 14 mm and a length of 1000 mm in annealed condition after prior plastic processing in the two-phase range with air cooling. The chemical composition of the alloy according to ISO 5832-11 was as follows (wt%): Al 5.5–6.5, Nb 6.5–7.5, Fe max. 0.25, C max. 0.08, H max. 0.009, O max. 0.2, rest Ti.

The test specimens, obtained in the form of 2 mm thick discs, were wet mechanically polished on silicon carbide abrasive papers of various grits in increasing gradations of 600, 1200, and 2500. Md-Mol polishing cloths and polishing suspensions (OP-S) were used for final polishing (Struers Inc., Cleveland, OH, USA). The polished samples were placed in an ultrasonic cleaner with ultrapure water (Milli-Q Advantage A10 Water Purification System, Millipore SAS, Molsheim, France) for 15 min, followed by acetone to remove undesirable contamination.

### 2.2. Production of ONTs on Ti–6Al–7Nb Alloy

For anodic oxidation, electrodes were made of polished alloy samples. An insulated copper wire was attached to the backside of the samples with epoxy resin, which provided electrical contact. The backside of the samples and the sides were protected with a chemically resistant, two-component epoxy resin. The production of self-assembling ONTs on the Ti–6Al–7Nb alloy with a geometric surface area of 1.5 cm^2^ was carried out using the anodic oxidation method at a constant voltage. The electrochemical modification of the alloy surface was carried out in a two-electrode system, in which the anode was an electrode from the tested alloy, while the cathode was a platinum mesh. The distance between the two electrodes was constant at 2 cm.

The anodic oxidation process was carried out using the PWR800H high-current power supply (Kikusui Electronics Corporation, Yokohama, Japan) at room temperature with the use of one type of weakly basic electrolyte. The electrolyte was an aqueous solution of 1 M EG with 0.2 M NH_4_F. Electrolytes were prepared using reagents of recognized analytical grade (Avantor Performance Materials Poland S.A., Gliwice, Poland). Anodic oxidation was carried out at a voltage (*E*) in the range of 5–100 V and a time (*t*) from 15 to 120 min. Each anode, after completion of the anodic oxidation process, was placed for 5 min in an ultrasonic cleaner with ultrapure water.

### 2.3. Physicochemical Characteristics of ONTs

Observations of the surface morphology and fractures of the Ti–6Al–7Nb alloy samples before and after anodic oxidation were carried out using the Supra 35 Scanning Electron Microscope (SEM, Carl Zeiss, Oberkochen, Germany). The tests did not require pre-treatment of the samples. Surface chemical composition analysis was performed using SEM in combination with Energy Dispersion X-ray Spectroscopy (EDAX) detector. High-resolution and precise imaging of the tested materials was ensured by a highly efficient In-lens Secondary Electrons (SE) detector.

X-ray Photoelectron Spectroscopy (XPS) was used to determine the type, amount, and chemical state of the elements present on the Ti–6Al–7Nb surface before and after anodizing. The measurements were performed using the PHI 5700 X-ray photoelectron spectrometer by Physical Electronics (ULVAC, Inc., Chigasaki, Japan). A beam of X-rays produced with an aluminum anode (monochromatic beam with an energy of 1486.74 eV) was used for the tests. The lamp power was 240 W (*U* = 15 kV, *I* = 16 mA). The tests were carried out in ultrahigh vacuum conditions of ~3 × 10^−9^ mbar. The diameter of the round area of analysis was 800 µm. High-resolution photoelectron spectra were analyzed using Multipak 9.4 software (ULVAC, Inc., Chigasaki, Japan). Carbon adsorbed on the surface of the samples, C1 peak (BE = 284.8 eV), was used to calibrate the spectra. Line deconvolution was implemented using a Shirley-type background cutoff and a Gaussian–Lorentzian line shape. 

### 2.4. Gentamicin Sulfate Loading and Release from ONTs on Ti–6Al–7Nb Alloy

ONTs on a Ti–6Al–7Nb alloy were tested as GS carriers with the molecular formula of C_19–21_H_39–43_N_5_O_7_·2.5H_2_SO_4_ (Merck KGaA, Darmstadt, Germany). A solution of 1% (*w*/*v*) GS in ultrapure water was prepared. Then, 100 µL of GS solution was pipetted onto the surface of the alloy with the formed ONTs and allowed to air dry. After 1 h, the samples were rotated and the operation was repeated to load as many ONTs as possible with the drug. Loading and drying steps were repeated 20 times to load a substantial amount of GS into the ONTs.

Thermogravimetry (TG), derivative thermogravimetry (DTG), differential thermal analysis (DTA), and evolved gas analysis (EGA) were performed using the thermal analyzer STA 409PC (NETZSCH, Selb, Germany), which was coupled with the quadrupole mass spectrometer QMS 403 C Aëolos (NETZSCH, Selb, Germany). To conduct the analysis, the sample was placed in an alumina crucible and heated within a range of temperature of 40–800 °C. The heating rate of the sample was 20 °C min^−1^. To prevent sample oxidation, testing was performed in argon with a flow rate of 50 mL min^−1^. TG analysis was performed to determine the amount of drug loaded in the ONTs. To find the temperature range and quantity of mass change during the drug decomposition, 16 mg of GS was placed in an alumina crucible and analyzed. Next, the GS loaded onto ONTs was analyzed and its amount was calculated from mass change quantity within a temperature range of 205–400 °C.

The release kinetics of GS implemented into ONTs was tested by immersing the sample in 15 mL of Phosphate Buffer Solution (PBS) at pH = 7.4(1) at 37 °C for 24 h. In the first 6 h, the PBS was collected for analysis every hour, then every 24 h for 7 days. Each time, 1 mL of PBS was collected and a fresh solution was added. The amount of released drug substance from ONTs was determined by UV–Vis spectroscopy (Biochrom WPA Biowave II UV/Visible Spectrophotometer, Cambridge, England). The absorbance was measured at the wavelength *λ* = 245 nm, determining the PBS absorbance value in the first step, and then the sampled solution. The amount of GS released in weight percentage was determined from the calibration curve for the drug.

## 3. Results and Discussion

### 3.1. SEM Studies of Microstructure

**Figure 1:**
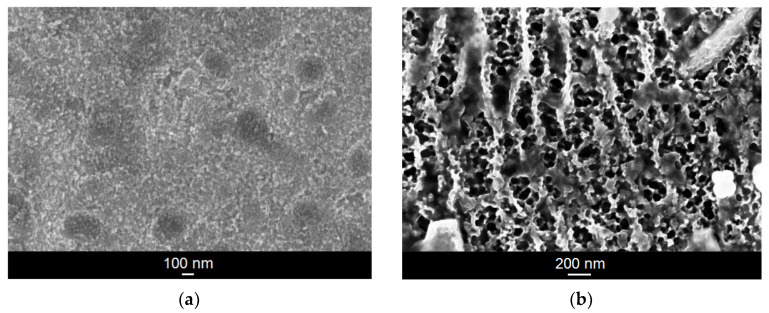
SEM image of the microstructure of the oxide layer formed on the Ti–6Al–7Nb alloy in 1 M EG + 0.2 M NH_4_F electrolyte under anodizing conditions: (**a**) 5 V for 120 min; (**b**) 100 V for 15 min.

The microstructure of the Ti–6Al–7Nb alloy after anodic oxidation in 1 M EG + 0.2 M NH_4_F electrolyte was characterized based on SEM images (Figure 1). In the case of a low voltage of 5 V, the nanotubular structure was not obtained despite the fairly long period of anodic oxidation (Figure 1a). However, the presence of spherical pores (darker areas) can be observed, the diameters of which range from 145 to 333 nm. Based on the SEM results obtained, it can also be concluded that very high voltages (approx. 100 V) with a short anodization time do not lead to the formation of single pores, as was the case with the extension of the oxidation time at low voltage. Instead, a very irregular and porous oxide layer is formed (Figure 1b).

**Figure 2:**
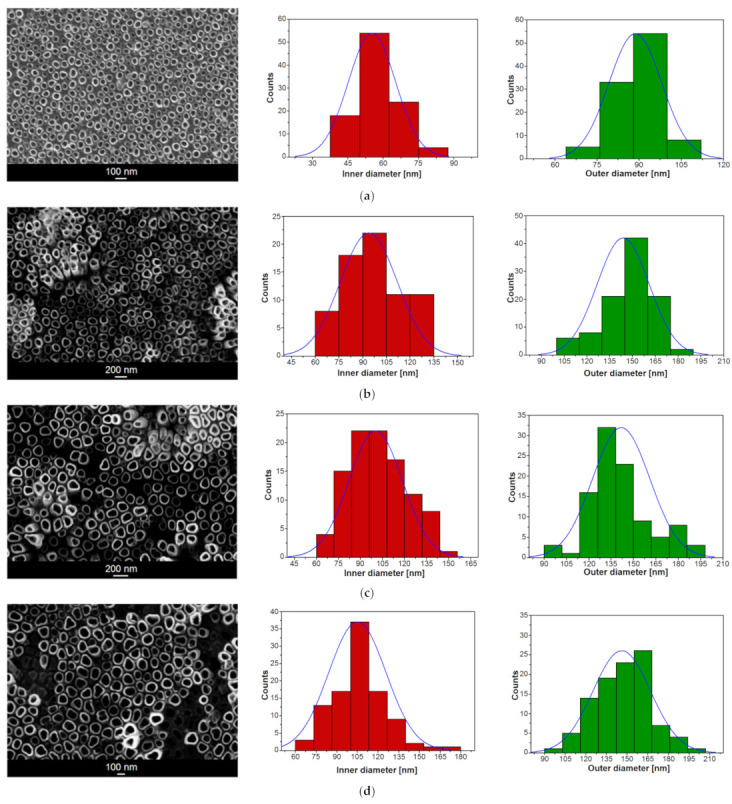

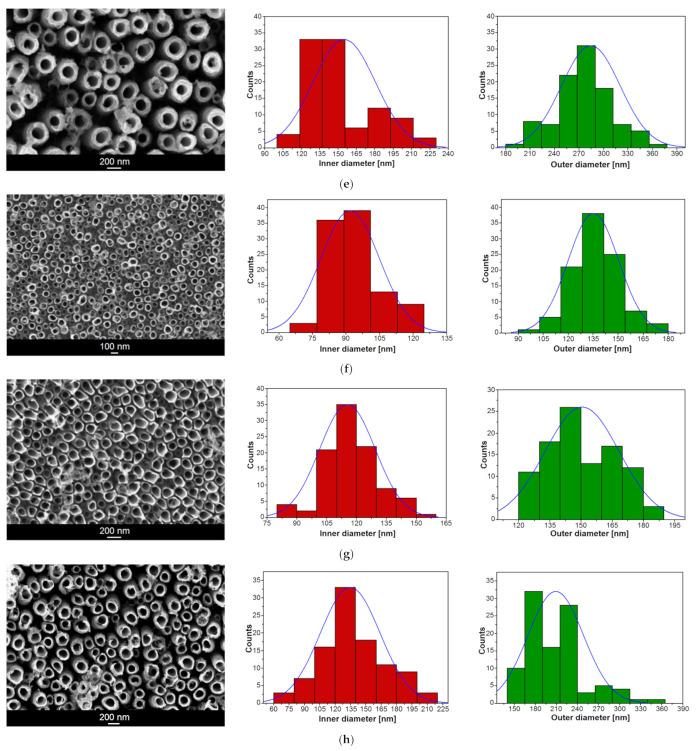
SEM image of the microstructure of oxide nanotube (ONT) layer formed on the Ti–6Al–7Nb alloy in 1 M EG + 0.2 M NH_4_F electrolyte under anodizing conditions with the corresponding histogram of inner and outer diameter distributions of ONTs: (**a**) 15 V for 60 min; (**b**) 30 V for 60 min; (**c**) 50 V for 60 min; (**d**) 70 V for 60 min; (**e**) 100 V for 60 min; (**f**) 30 V for 45 min; (**g**) 50 V for 30 min; (**h**) 70 V for 20 min. Gaussian fitting curve is marked as blue solid line.

The SEM images in Figure 2 presenting the on-top general view of the Ti–6Al–7Nb alloy after anodic oxidation in 1 M EG + 0.2 M NH_4_F electrolyte at voltages ranging from 15 to 100 V and with an anodizing time from 20 to 60 min show the oxide layer microstructure with the parallel arrangement of single-walled ONTs. Generally, an even distribution of ONTs can be observed. However, in some cases, randomly distributed bundles consisting of packed ONTs are present (Figure 2b,c). It is assumed that ONT bundles grew in local micro-areas where surface pitting initially occurred. Based on the SEM results obtained, it can be concluded that higher voltages generate ONTs of regular shapes. As the anodizing voltage increased in the range of 70–100 V, ONTs’ brittleness and the formation of insoluble titanium deposits on the surface of nanotubular oxide layers became apparent.

The passive surface layer on titanium and its alloys contain titanium in the three oxidation states Ti^4+^, Ti^3+^, and Ti^2+^ [3,25,26,27]. The Ti^4+^ ions are formed the most frequently, which combine with O^2−^ and OH^−^ to form insoluble complexes.

**Figure 3:**
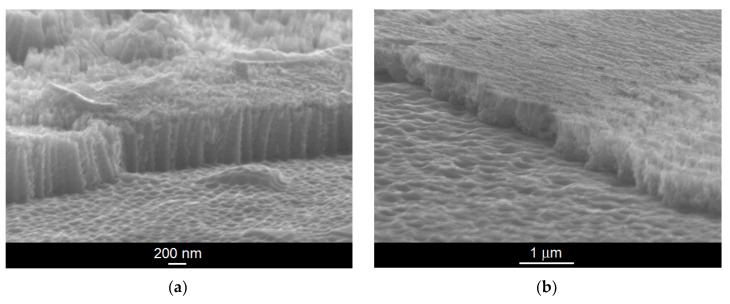
SEM image of the fracture of oxide nanotube (ONT) layer formed on the Ti–6Al–7Nb alloy in 1 M EG + 0.2 M NH_4_F electrolyte under anodizing conditions: (**a**) 30 V for 45 min; (**b**) 70 V for 20 min.

Exemplary SEM images of mechanically scratched regions of the ONT layer formed on the Ti–6Al–7Nb alloy in 1 M EG + 0.2 M NH_4_F electrolyte under anodizing conditions at 30 V for 45 min and 70 V for 20 min are shown in Figure 3a,b, respectively. The SEM images show a top view of the mechanically fractured samples with visible and invisible areas of the ONTs. Thin oxide layers in the form of an ordered matrix built of vertically oriented nanotubes can be seen. The obtained ONTs present the highest degree of ordering and are characterized by smooth walls, which will ensure high resistance to corrosion in the biological environment. This is of great importance in medical applications where corrosion resistance is essential [7,10,14,18].

The morphological parameters of the ONTs formed on the Ti–6Al–7Nb alloy in 1 M EG + 0.2 M NH_4_F electrolyte, such as the inner (*D*_i_) and outer (*D*_0_) diameters of the nanotube and the length of the nanotube (*L*), were determined based on SEM images from selected surface areas (see Figure 2 and Figure 3). For all investigated ONT layers, empirical histograms of *D*_i_ and *D*_0_ distributions were prepared. The average values of *D*_i_ and *D*_0_ were estimated from a Gaussian fit to the diameter histogram. The relationship between the morphological parameters of the obtained ONTs and the voltage–time conditions of the anodizing process is presented in Table 1.

Based on the obtained results, it can be concluded that both the applied voltage and the time of anodic oxidation play an important role in the formation of the third-generation ONT layers on the Ti–6Al–7Nb alloy surface and in shaping their morphologies. With an increasing voltage in the range of 15–100 V for 60 min of anodizing, the average *D*_i_ of the obtained ONTs increases in the range from 45(5) to 155(25) nm and the average *D*_0_ from 69(8) to 285(36) nm. The longest ONTs of *L* = 10.85(25) µm were achieved at a voltage of 50 V for an oxidation time of 60 min, while the shortest ones of *L* = 0.28(1) µm were formed at the same voltage during 30 min. At the highest voltage of 100 V for 60 min, the *L* of ONTs was 2.40(24) µm. For comparison, at a voltage of 70 V and a much shorter time, i.e., 20 min, ONTs with *L* = 0.50(4) µm were formed. For a voltage of 30 V and an anodizing time of 45 min, ONTs long at 0.75(1) µm were obtained. The longer the anodizing time, the longer the ONTs obtained, taking into account the voltage, which cannot be too high, because at high voltages we observe an increase in the *D*_i_ and *D*_0_ of the ONTs and not their *L*.

The total surface area (*A_i_*) of the ONT was calculated according to Equation (1) [28]:

(1) $$A_{t}=2\pi (D_{0}^{2}-D_{i}^{2})+2\pi L(D_{0}+D_{i}).$$

The specific surface area (*A_s_*) of the ONT expressed in cm^2^ per 1 cm^2^ was estimated using Equation (2) [28]:

(2) $$A_{s}=n\cdot A_{t},$$

where *n* is a total number of ONTs occurring on the surface area of 1 cm^2^.

One can see that the highest *A*_s_ of 600 cm^2^ per 1 cm^2^ is determined for the ONT layer formed at 50 V for 60 min of anodizing. The use of lower and higher voltages with an anodizing time of 60 min allows one to obtain ONTs characterized by *A*_s_ with an almost two times lower value. Reducing the anodizing time to less than 60 min leads to a drastic reduction in *A*_s_ (Table 1). For comparison, the *A*_s_ of only 49.4 cm^2^ cm^−2^ was evaluated for the ONT layer formed on the Ti–13Zr–13Nb alloy during anodizing in 1 M EG electrolyte with 4 wt% content of NH_4_F at 50 V for 80 min [10]. In organic-based electrolyte containing EG + 0.3 M NH_4_F + 0.2 M H_3_PO_4_ + 0.15% H_2_O, the *A*_s_ of about 77 cm^2^ cm^−2^ was found for Ti after anodic oxidation at 20 V for 120 min [28].

The geometry of the third-generation ONTs obtained on the Ti–6Al–7Nb alloy under the proposed conditions can be easily tailored in a controlled manner depending on the particle size of the drugs and the clinical dose required for individual patients. Taking into account the *A*_s_ value, the optimal conditions of anodic oxidation are achieved at 50 V for 60 min. The ONTs formed at these operating parameters ensure the highest drug-loading capacity. The proposed functionalization of the Ti–6Al–7Nb alloy surface enables self-assembling, nanotubular oxide structures characterized by a wide range of geometries. Morphologically, the obtained ONTs are very similar to the structure of bone tissue. They ensure an increase in the surface roughness of the alloy at the nanoscale, thus contributing to the improvement of the osseointegration process through the faster growth of bone tissue and stronger bonding to the implant. The resulting nanostructures may provide better osteoblast interactions as the adsorption of proteins that mediate osteoblast adhesion, such as vitronectin and fibronectin, are released on nanophasic substances. The obtained ONTs can be proposed for use in modern implantology as a drug delivery system for various therapeutic purposes [12,13,14,15,16,18,19,20,21,22,23,24,25,26,27,28,29].

### 3.2. EDAX Study of Chemical Composition

**Figure 4:**
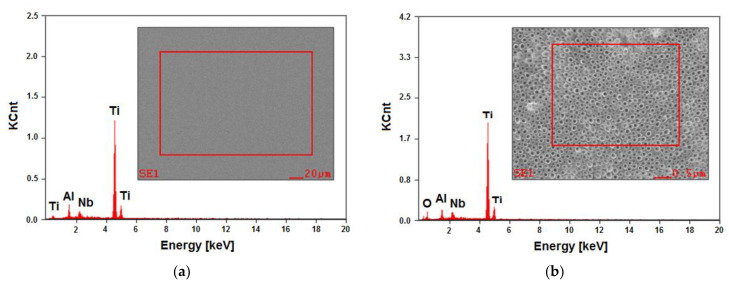
EDAX spectrum with a selected micro-area for analysis from the Ti–6Al–7Nb alloy surface: (**a**) before anodizing; (**b**) after anodizing at 15 V for 60 min.

The material used as the substrate for the anodic oxidation was a commercial alloy of Ti–6Al–7Nb (wt%). Such a chemical composition provides appropriate properties for applications in the field of medical implants, additionally ensuring greater biocompatibility and corrosion resistance compared to titanium and its alloys containing vanadium in its composition [12,13,14,15,16,17,18,19,20,21]. The control analysis of the chemical composition of the tested alloy was carried out using the EDAX method, which allowed for quantitative and qualitative analyses of the chemical composition in micro-areas (Figure 4a). The composition of each sample after anodic oxidation was studied using the EDAX method. The composition of Ti–6Al–7Nb after the anodic oxidation whilst varying the anodization parameters was similar. The exemplary results obtained for the sample oxidized at 15 V for 60 min are presented.

The EDAX spectrum, being dependent on the number of counts in the function of radiation energy, showed the presence of peaks originating from the substrate, i.e., Ti, Al, and Nb, both for the Ti–6Al–7Nb alloy in the initial state (Figure 4a) and after anodizing at 15 V for 60 min (Figure 4b). The surface contents of elements determined based on the peaks identified for the alloy before electrochemical modification were found to be 86.0(2) wt%. % for Ti, 6.7(4) wt% for Al, and 7.3(3) wt% for Nb. The analysis of the obtained results did not reveal any discrepancies concerning the requirements specified for the Ti–6Al–7Nb alloy in the ISO 5832-11 standard. The presence of an additional peak from oxygen in the EDAX spectrum for the anodized alloy showed that an oxide layer was present on the surface of the tested material (Figure 4b).

Reflectometric studies have shown that the obtained ONTs on the Ti–6Al–7Nb alloy surface are amorphous regardless of the anodizing parameters [30]. The beam incidence angle did not exceed a few degrees and ranged from 0.2 to about 1.0°. The exemplary grazing incidence X-ray diffraction pattern recorded in the range of 2*θ* angles from 10 to 90° for the alloy oxidized at 15 V for 60 min showed reflections from the two-phase (α + β) substrate and amorphous “halo” at the 2*θ* angles from 15 to 30°. As the angle of incidence decreases, the amorphous “hallo” becomes more visible, although it is invisible at α equal to 1.

### 3.3. XPS Study of Chemical States

**Figure 5:**
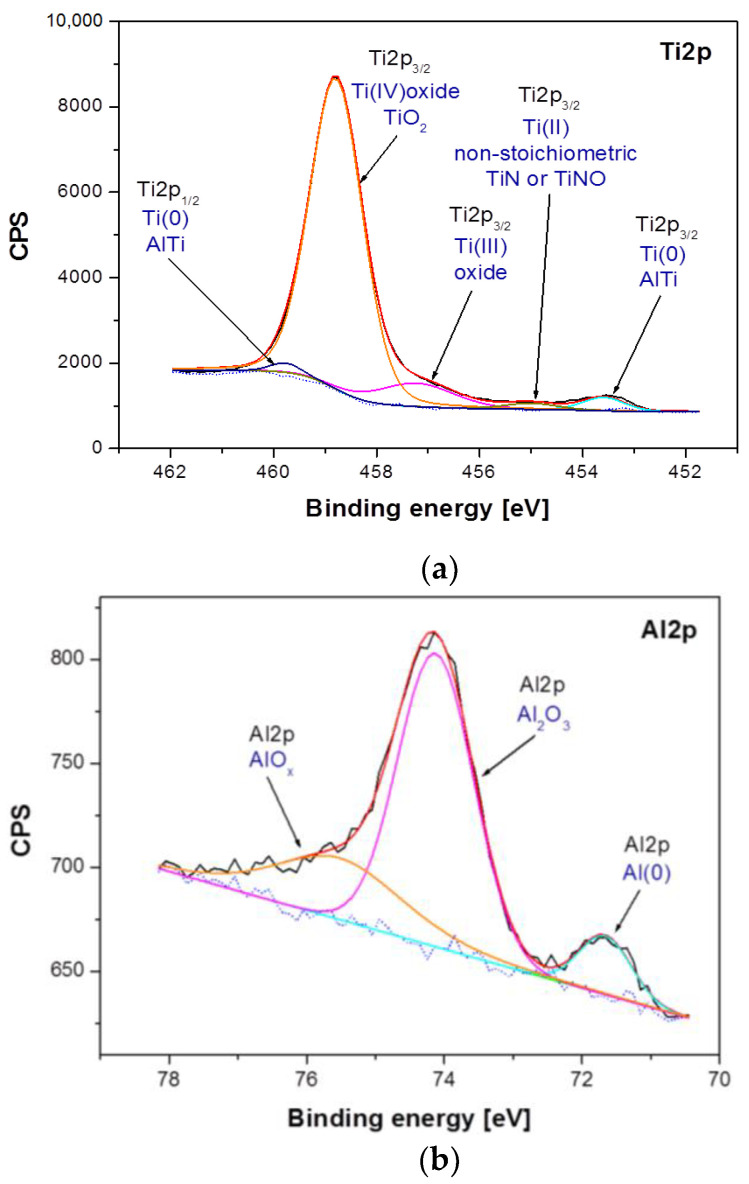

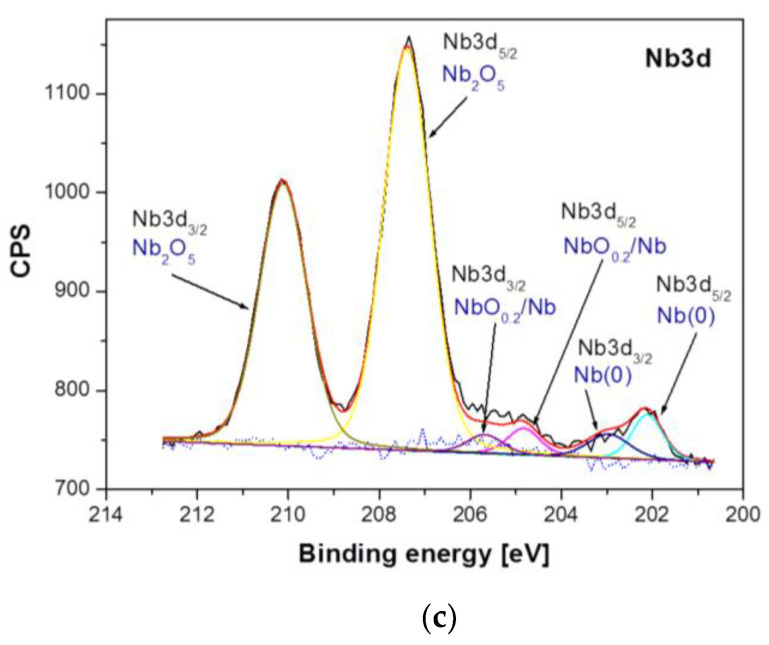
High-resolution XPS spectra for the photoemission line: (**a**) Ti2p; (**b**), Al2p and (**c**) Nb3d, obtained for the Ti–6Al–7Nb alloy in the initial state.

**Figure 6:**
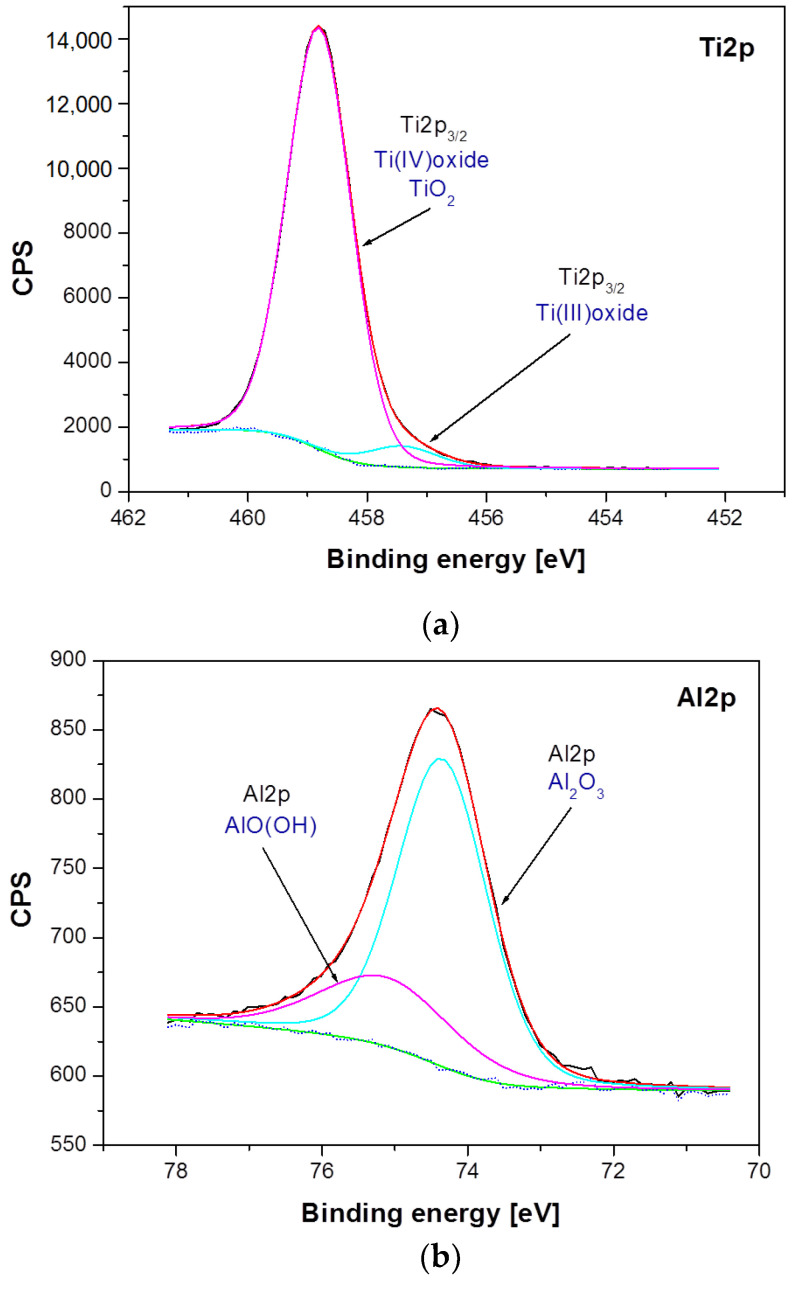

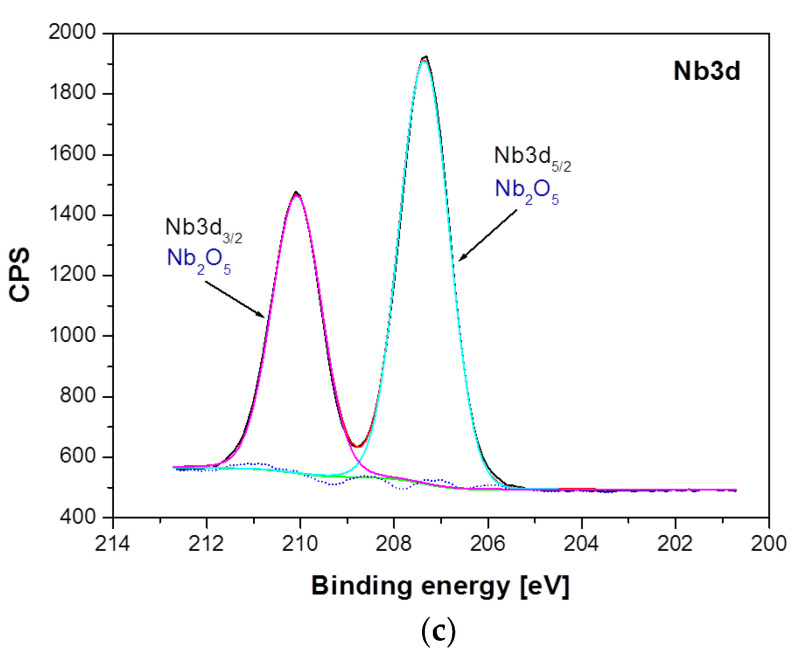
High-resolution XPS spectra for the photoemission line: (**a**) Ti2p; (**b**), Al2p and (**c**) Nb3d, obtained for the Ti–6Al–7Nb alloy after anodic oxidation at 15 V for 60 min.

The XPS analysis focused on determining the chemical states of the tested components of the Ti–6Al–7Nb alloy before and after anodic oxidation (Figure 5 and Figure 6). As a result of irradiating the alloy surface with a beam of monochromatic X-ray radiation, electrons were emitted from a depth of 3–4 nm. The kinetic energy of the emitted photoelectrons was measured, from which the binding energy was then determined. The binding energy is characteristic of a given element and its chemical state, which made it possible to determine chemical compounds or bonds occurring between the elements of the research material according to Equation (3) [31]:

*E*_B_ = hν − *E*_k_ − *Φ*, (3)

where hν = 1486.74 eV is X-ray energy, *E*_k_ is the kinetic energy of the broken away photoelectron (measured value), *Φ* denotes the spectrometer function taking into account the work of the electron output from the sample and *E*_B_ is the binding energy determined from the measurements and subject to analysis. The analysis of high-resolution photoemission spectra was limited to the basic elements included in the characterized samples.

Differences between samples have been shown both in the atomic concentration and in the presence of individual chemical states of the sample components. The analysis of atomic concentration, numerical data given for calculations including Ti, Al, Nb, O, and N, indicates number of differences between samples. The Ti–6Al–7Nb alloy after electrochemical modification contains less nitrogen (1.84 atomic percent, at. %), oxygen (69.83 at. %) and aluminum (3.95 at. %) on the surface in comparison with the Ti–6Al–7Nb alloy before the modification (N: 3.4 at. %, O: 71.37 at. %, Al: 4.33 at. %). An increase in atomic concentration due to electrochemical modification was, however, observed for titanium (from 19.88 at. % before modification up to 22.49 at. % after modification) and niobium (from 1.01 at. % to 1.9 at %). Such variations may occur partly due to different amounts of surface contamination and to the surface modification process itself. These variations were accompanied by changes in the chemical states of the individual elements visualized in the high-resolution XPS spectra in Figure 5 and Figure 6. Figure 5 shows a detailed analysis of Ti2p, Al2p and Nb3d photoemission lines for the Ti–6Al–7Nb alloy before electrochemical modification and Figure 6 after modification.

Analysis of the XPS spectra of the Ti–6Al–7Nb alloy in the initial state presented in Figure 5 showed that titanium exists in four chemical states: (i) Ti(0): AlTi Ti2p_3/2_ line position at 453.60 eV [32]; (ii) Ti(II): non-stoichiometric TiN or TiNO Ti2p_3/2_ line position at 455.00 eV [33,34]; (iii) Ti(III): Ti2p_3/2_ oxide line position at 457.22 eV [35]; (iv) Ti(IV): TiO_2_ oxide Ti2p_3/2_ line position at 458.78 eV [35] (Figure 5a). The analysis of the Al2p line shape of the Ti–6Al–7Nb alloy before electrochemical modification indicates the presence of aluminum in three chemical states: (i) Al(0)—metallic bond for Al2p at 71.66 eV; (ii) Al_2_O_3_ for the Al2p baseline at 74.13 eV [36], and (iii) AlO_x_ for Al2p at 75.53 eV [37] (Figure 5b). In the case of niobium, three chemical states were detected: (i) Nb(0)—metallic bond for the Nb3d_5/2_ line position at 202.11 eV and Nb3d_3/2_ at 202.96 eV; (ii) NbO_0.2_/Nb for the Nb3d_5/2_ baseline at 204.83 eV [38] and Nb3d_3/2_ at 205.68 eV, and (iii) Nb_2_O_5_ for the Nb3d_5/2_ line position at 207.39 eV [39] and Nb3d_3/2_ at 210.11 eV (Figure 5c). 

Figure 6 presents a detailed analysis of Ti2p, Al2p, and Nb3d photoemission lines for the Ti–6Al–7Nb alloy after anodic oxidation at 15 V for 60 min. The analysis of the obtained XPS spectra confirmed the presence of titanium in two chemical states: (i) Ti(IV) TiO_2_ oxide for the Ti2p_3/2_ line position at 458.81 eV [35], and (ii) Ti(III) non-stoichiometric oxide for the Ti2p_3/2_ baseline at 457.42 eV (Figure 6a). The position and shape of the Al2p line of the anodized Ti–6Al–7Nb alloy indicate the presence of aluminum in two chemical states: (i) Al_2_O_3_ for the Al2p baseline at 74.35 eV [40], and (ii) AlO(OH) for the Al2p line position at 75.12 eV (Figure 6b). The position and shape of the Nb3d line for the Ti–6Al–7Nb alloy after an electrochemical modification indicate the presence of niobium in only one chemical state, Nb_2_O_5_, which has been identified based on the position of the Nb3d_5/2_ line position at 207.4 eV [41] (Figure 6c).

Among the differences observed between the samples, the disappearance of chemical states associated with the metallic states of Ti, Nb, and Al is the most pronounced. The electrochemical modification also leads to the disappearance of titanium and niobium in the divalent state. Considering that electrochemical modification was carried out in aqueous media, it can be concluded that titanium and niobium in such an environment preferably bond with oxygen, most likely from water, forming TiO_2_ and Nb_2_O_5_ compounds. The modification of the aluminum oxide structure present in the reference sample leads to the formation of an additional bond with the OH group. The observed differences are accompanied by a change in the chemical state of oxygen. A decay of the high-binding energy states of oxygen (O1s peaks in the range 531–533 eV associated with organic compounds appearing due to atmospheric surface contamination or C–Ti–O_x_ and C–Ti–(OH)_x_ similarly as presented in [42]) was observed after electrochemical modification. 

### 3.4. Mechanism of ONT Formation on Ti–6Al–7Nb Alloy 

**Figure 7:**
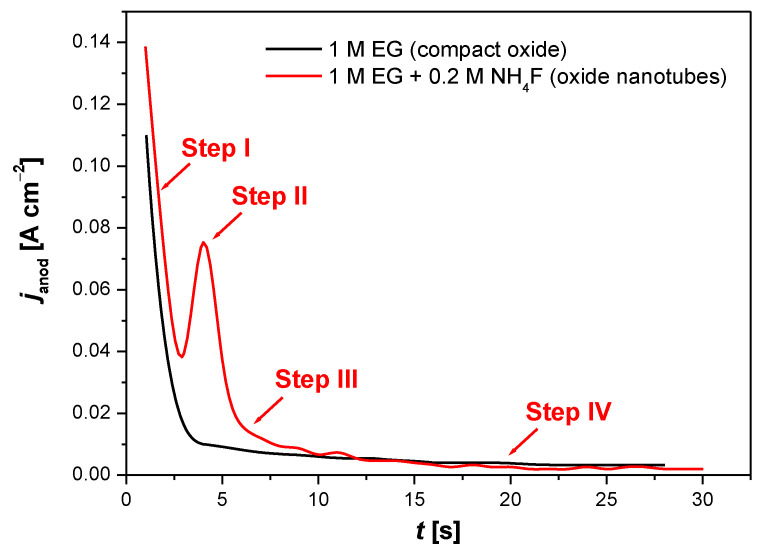
The curve of anodic current density (*j*_anod_) as a function of time (*t*), recorded during the initial anodization stage for Ti–6Al–7Nb electrode at 30 V for 60 min in 1 M ethylene glycol (EG) electrolyte in the presence (**—**) and absence (**—**) of 0.2 M NH_4_F.

The formation of the oxide layer on the Ti–6Al–7Nb alloy can be monitored by recording current–time characteristics. Figure 7 displays the course of an exemplary curve showing the dependence of the anode current density (*j*_anod_) as a function of time, recorded at the initial stage of the electrochemical oxidation of Ti–6Al–7Nb electrode at 30 V for 60 min in 1 M EG electrolyte without (black line) and with (red line) fluoride ions. 

In the absence of fluoride ions, the obtained oxide layer was uniform and compact (Figure 7, black line). The anodic oxidation of metal (Me) in a GE-based electrolyte without fluoride ions proceeded according to Reactions (4)–(7), where Me = Ti, Al, Nb [14,15,25,26,27]:

(4) $$\mathrm{Me}\to {\mathrm{Me}}^{n+}+{\mathrm{ne}}^{-},$$

(5) $$\mathrm{Me}+\frac{n}{2}H_{2}O\to {\mathrm{MeO}}_{n/2}+{\mathrm{nH}}^{+}+{\mathrm{ne}}^{-},$$

(6) $${\mathrm{Me}}^{n+}+{\mathrm{nH}}_{2}O\to {\mathrm{Me}(\mathrm{OH})}_{n}+{\mathrm{nH}}^{+},$$

(7) $${\mathrm{Me}(\mathrm{OH})}_{n}\to {\mathrm{MeO}}_{n/2}+\frac{n}{2}H_{2}O.$$

The process of hydrogen electroevolution, described by Reaction (8), took place on the cathode [25,27]:

(8) $${\mathrm{nH}}_{2}O+{\mathrm{ne}}^{-}\to \frac{n}{2}H_{2}↑+{\mathrm{nOH}}^{-}.$$

The formation of a thin oxide layer was initiated by the reaction of oxidized Ti, Al, and Nb with O^2−^ and OH^−^ ions coming from water. Further growth of the anodic layer was controlled by several processes, among which the high-filed ion migration of Me^n+^ and O^2−^ through the formed oxide layer played the most important role. Me ions migrate towards the electrolyte, while O^2−^ ions towards the alloy surface, where they react with its constituent metals. The process of dissolving the oxide also affects the thickness and structure of the obtained oxide layer. The dissolution can be caused by the presence of an electric field that weakens the Me–O bonds, or it can be purely chemical in nature due to the composition of the electrolyte [14,15,25,26,27]. In the case of constant voltage anodization, the electric field strength decreases as the oxide layer thickens, which is experimentally observed as an exponential drop in current. The oxide layer growth process slows down spontaneously, which results in the obtained anodic layer having a finite thickness. The oxide layer growing at the oxide/electrolyte interface is less dense and contains oxyhydroxides, while the layer formed at the metal/oxide interface is composed of a dense and stable oxide. In the case of titanium, the oxide increases at the metal/oxide interface [25]. 

The electrochemical formation of ONTs on the Ti–6Al–7Nb electrode surface took place only in the EG electrolyte in the presence of fluoride ions (Figure 7, red line), which confirms the presence of a typical trough on the *j*_anod_ = f(*t*) curve [25,26,27]. F^−^ ions have the ability to form water-soluble complexes with Ti, Al, and Nb following Reactions (9)–(11) [15,25]:

(9) $${\mathrm{TiO}}_{2}+6F^{-}+6H^{+}\to {\left[{\mathrm{TiF}}_{6}\right]}^{2-}+2H_{2}O,$$

(10) $${\mathrm{Al}}_{2}O_{3}+6F^{-}+6H^{+}\to {2\mathrm{AlF}}_{3}+3H_{2}O,$$

(11) $${\mathrm{Nb}}_{2}O_{5}+12F^{-}+10H^{+}\to 2{\left[{\mathrm{NbF}}_{6}\right]}^{-}+5H_{2}O.$$

In the presence of an electric field, F^−^ ions can migrate deep into the oxide layer formed competing with O^2−^ ions and cause its local dissolution. At the same time, F^−^ ions inhibit the deposition of titanium hydroxides on the oxide layer by complexing Ti^4+^ ions migrating to its surface, which are ejected at the oxide/electrolyte interface according to Reaction (12) [15,25]:

(12) $${\mathrm{Ti}}^{4+}+6F^{-}\to {\left[{\mathrm{TiF}}_{6}\right]}^{2-}.$$

Then, Reaction (13) takes place, releasing oxygen from the water [15,25]:

(13) $$2H_{2}O+{4e}^{-}\to O_{2}+{4H}^{+}$$

The process of the electrochemical production of the ONT matrix on the Ti–6Al–7Nb alloy surface can be divided into four steps (Figure 7, red line). The course of the *j*_anod_ = f(*t*) curve at the initial anodization is similar to the current–time characteristic in the fluoride-free electrolyte (Figure 7, black line). In step I, which is the shortest, a rapid and exponential decrease in the *j*_anod_ value is observed, which is related to the increase in the thickness of the compact oxide layer produced. In step II, the increase in the *j*_anod_ value occurs, which is related to the chemical dissolution of the oxide layer in the presence of the fluoride anions in Reactions (9)–(11). There is a formation of irregular nanoscale pores and a reduction in the thickness of the oxide barrier layer at the bottom of the pores. The *j*_anod_ increases as a result of the growth of the reactive surface area. In step III, the *j*_anod_ drops due to the formation of a regular nanopore or nanotube layer. In step IV, an equilibrium is established between the oxidation and dissolution process with the formation of a nanostructured surface, and the *j*_anod_ becomes time-independent. 

**Figure 8:**
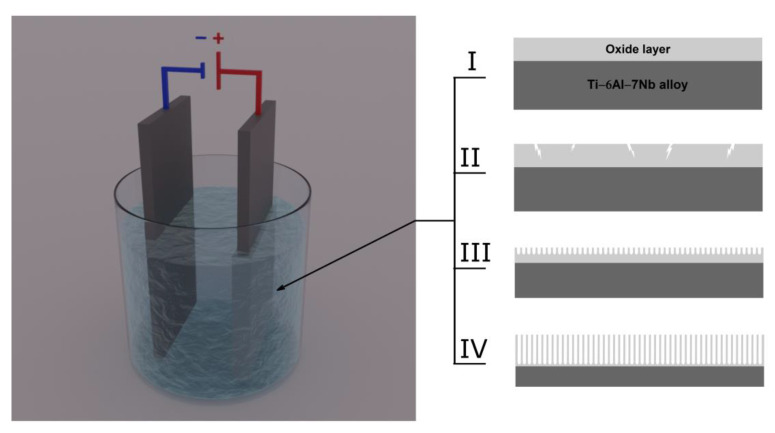
Diagram illustrating the multistep mechanism of oxide nanotube (ONT) formation on Ti–6Al–7Nb alloy during anodization in an aqueous solution of ethylene glycol containing fluoride ions: (Step I) formation of a compact oxide layer; (Step II) formation of pores; (Step III) formation and separation of ONTs; (Step IV) determination of the length of ordered ONTs.

A schematic diagram illustrating the multistep mechanism of ONT formation on the Ti–6Al–7Nb alloy during anodization in an aqueous solution of EG containing fluoride ions is shown in Figure 8. Anodization, which is an electrolytic process that can be used to produce ONT layers on the surface of the tested alloy, was carried out with the use of an external power source. The anode was oxidized alloy, which was placed face to face with the cathode in the electrolyte. The cathode is typically used in the form of a platinum plate, although carbon cathodes may also be found to be in use. A current with the desired parameters was passed through the two-electrode system, and by transferring the charge on the electrode/electrolyte interface, the substrate dissolved and a porous surface was obtained. Depending on the value of the voltage on the anode surface, a continuous oxide layer with a thickness and structure that change with increasing voltage, a porous layer, or a layer of ONTs can be formed. 

The mechanism of ONT formation on the Ti–6Al–7Nb alloy can be based on four characteristic steps distinguished in the anodic oxidation process. In step I, a compact oxide barrier layer is formed, which tightly adheres to the anode surface and reduces the current density due to the low conductivity of the oxides. In the next anodizing step, narrow gaps and cracks appear on the anode surface due to the dissolution of the oxides (Step II). The density of the oxides begins to increase. In stage III, the current density reaches a relatively constant value, and pores are formed in the cracks and crevices. As the anodizing time is extended, the pores bifurcate, begin to overlap, and compete for the available current. The ONT matrix is shaped in optimal current–voltage conditions, in which the current is distributed evenly between the pores, leading to the self-reordering of the porous oxide layer. The ONT matrix is shaped in optimal current–voltage conditions, which ensure an even distribution of the current between the pores, leading to self-organization of the porous oxide layer. The most important factors affecting the geometry of ONTs include the type of electrolyte, anodizing voltage–time conditions, and the content of water and F^−^ ions in the electrolyte [6,7,8,9,10,12,13,14,15,16,18,19,20,21,23,24,25,26,27]. In step IV, the rate of oxidation of titanium and alloying elements is equalized with the rate of dissolution of the formed metal oxides by F^−^ ions. The thickness of the barrier oxide layer at the bottom of the ONTs and in the space between them remains constant. Increasing the length of ONTs can be treated as a fourth step of the entire anodizing process or as an extension of step III. The growth rate of ONTs gradually decreases during anodization. After it is equal to the rate of chemical dissolution of the upper surface of the ONT layer, continuing the anodic oxidation process does not increase the length of the produced ONTs [5,25].

### 3.5. Kinetics of Gentamicin Sulfate Release from ONTs 

The inflammation that develops in bone tissue after implantation causes the multiplication of bacteria and requires immediate local action of the drug substance. A delay in the administration of a drug, in particular an antibiotic, may increase the growth of bacteria in the form of a bacterial biofilm and cause inflammation of the bone tissue. An inflamed state may result in the rejection of the implant and reoperation. It is therefore advantageous when the implants used have a built-in drug release function within the tissues in which they are embedded. The drug can release a larger therapeutic dose quickly, which will effectively protect the implant from rejection and infection. Such a system reduces the waste of the drug because the medicinal substance goes directly to the place that requires it [23,24].

**Figure 9:**
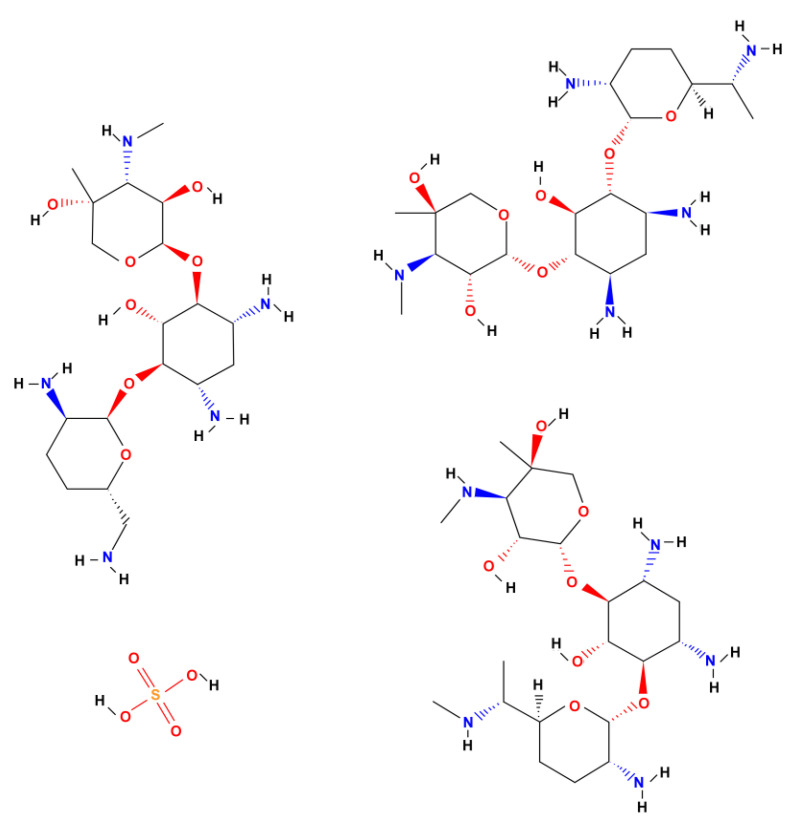
Chemical structure of gentamicin sulfate (GE).

The ONTs formed on the Ti–6Al–7Nb alloy in the optimal anodizing conditions at 50 V for 60 min in 1 M EG + 0.2 M NH_4_F electrolyte were selected as prospective drug carriers. The highest value of drug-loading capacity was expected for this ONT layer due to the greatest *A*_s_ of 600 cm^2^ cm^−2^ (Table 1). Gentamicin sulfate was used as a model antibiotic. The chemical structure of GE is presented in Figure 9.

GS is a broad-spectrum aminoglycoside antibiotic produced naturally by the actinomycete *Micromonospora purpurea* to combat bacterial infections. GS is used to treat serious bone tissue infections caused by the susceptible *Staphylococcus aureus*, *Citrobacter freundii*, *Enterobacter aerogenes*, *Escherichia coli*, *Klebsiella pneumoniae*, *Proteus mirabilis*, *Serratia marcescens*, or *Pseudomonas aeruginosa*. This drug acts mostly against Gram-negative bacteria by inhibiting the growth of the bacteria by limiting their synthesis. GS binds irreversibly to site A of the ribosome, preventing bacteria from incorporating new amino acids into the resulting protein. Its action depends on the penetration of the drug into the bacterial cell, in which the ribosomes are located. GS is water-soluble and partially insoluble in alcohol. The use of GE in controlled drug release systems will allow for targeted drug delivery directly to the tissues around the implant, which will positively affect the immune system’s response and inhibit the process of bacterial growth [43,44,45]. GS is currently on the World Health Organization’s (WHO) List of Essential Medicines [46]. The WHO classifies GS as critically important for human medicine. 

**Figure 10:**
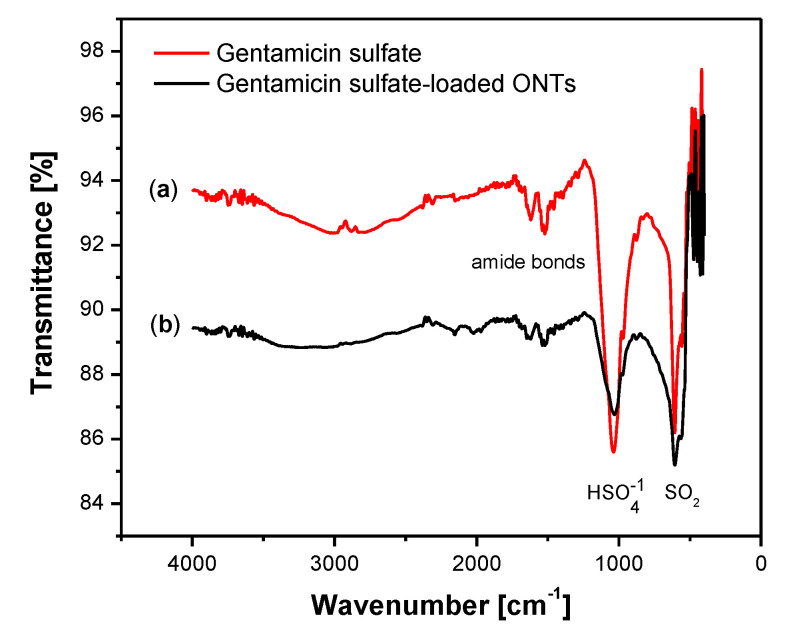
ATR-FTIR spectrum collected for: (**a**) gentamicin sulfate; (**b**) gentamicin sulfate–loaded ONTs formed on the Ti–6Al–7Nb alloy in 1 M EG + 0.2 M NH_4_F electrolyte anodized at 50 V for 60 min.

To prove the GS loading into the obtained ONTs, the ATR-FTIR spectrum was collected for GS in pure form and after loading into ONTs (Figure 10). The ATR-FTIR spectrum of GS shows typical absorption bands at 1616, 1558, and 1456 cm^−1^ which belong to the amide I, amide II, and gentamicin amide bonds [43]. The peak located at 1035 cm^−1^ is related to the ${\mathrm{HSO}}_{4}^{-1}$ group. The peak at 607 cm^−1^ is due to the SO_2_ band [44]. The ATR-FTIR spectrum obtained for ONTs with an implemented drug confirms the presence of GS in nanotubular oxide structures acting as a drug carrier. In ONTs loaded with GS, spectra at 1032 cm^−1^ and 607 cm^−1^ are visible, which proves the successful application of the drug to the ONTs formed on the Ti–6Al–7Nb alloy.

**Figure 11:**
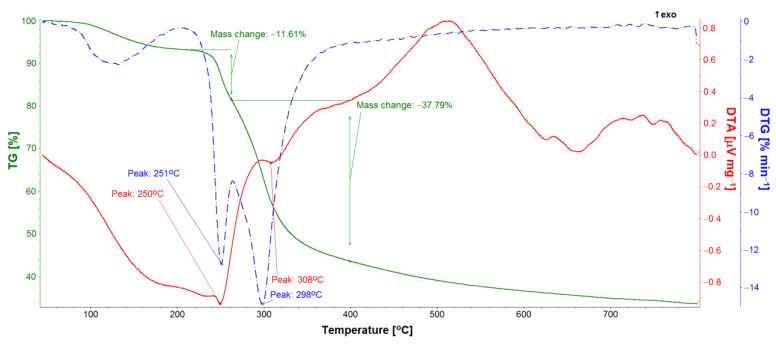
TG, DTA, and DTG curves of gentamicin sulfate sample.

**Figure 12:**
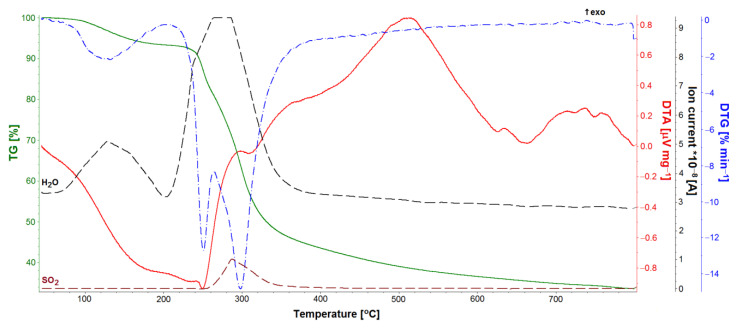
TG, DTA, and DTG curves and evolved gas analysis of gentamicin sulfate sample.

**Figure 13:**
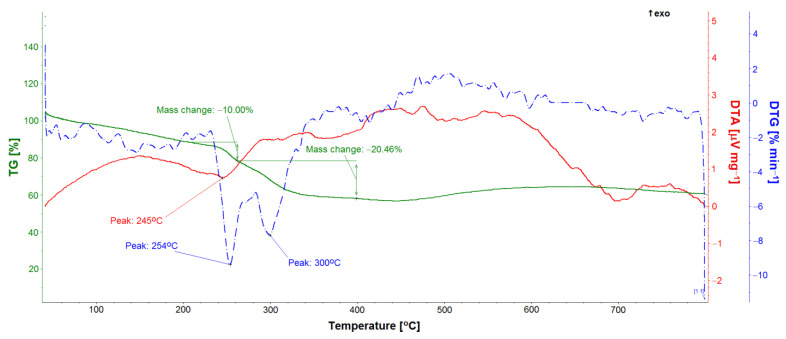
TG, DTA, and DTG curves of gentamicin sulfate-loaded ONTs.

TG, DTA, and DTG studies allowed for the quantitative analysis of the GS loaded into the ONTs (Figure 11, Figure 12 and Figure 13). Five GS-loaded ONTs with a total mass of 1.5 mg were analyzed. For both the GS sample and GS-loaded ONTs with a temperature range of up to 200 °C, physically adsorbed water release was observed (Figure 11 and Figure 12), as evidenced by the water line recorded on the mass spectrometer (Figure 12). For the temperature range 205–400 °C, mass loss (49.40% for GS and 30.46% for GS-loaded ONTs) due to the drug decomposition is visible on the TG curves. This is related to evolving water (Figure 12) and hydrocarbons weighing more than 90 u. Drug decomposition is a two-step process. The first stage ensures the maximum speed at 251 °C for the drug and 254 °C for drug-loaded ONTs (based on the DTG curve). The associated endothermic effects at 250 °C for drug and 245 °C for drug-loaded ONTs are also visible. At the second stage, sulfate decomposes what is detected in evolved gas analysis (Figure 12). The maximum speed of decomposition at this stage is observed at 298 and 300 °C for drug and drug-loaded ONTs, respectively. The endothermic effect at a temperature of 308 °C is also visible for the drug. The TG confirmed the successful loading of drug into ONTs with the loading amount at around 62% versus the total sample mass (a drug with ONTs).

**Figure 14:**
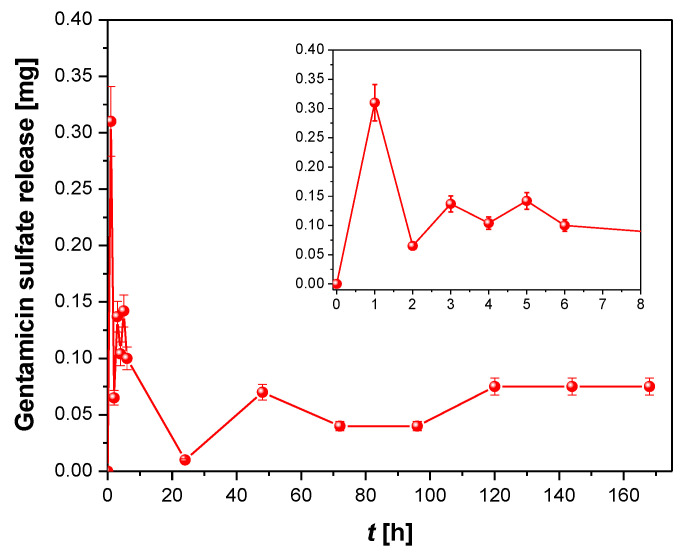
Gentamicin sulfate release from ONTs on the Ti–6Al–7Nb alloy as a function of immersion time in phosphate-buffered solution. The inset corresponds to the first 6 h of drug release.

To prove the drug-eluting ability of the obtained ONTs, GS release profiles from the nanotubes on the Ti–6Al–7Nb alloy, as a function of immersion time in PBS, were recorded (Figure 14). 

The kinetics of GS release can be divided into two steps. In the first step, illustrated by the inset in Figure 14, GS was bursty released from the interior of the ONTs in the first 6 h in an amount from 0.3 to 0.1 mg. Maximum drug ejection was achieved within the first hour. Such a drug release course indicates a rapid diffusion of loosely bound GS molecules at the top of the ONTs due to the high concentration gradient between the GS interface at the ONT layer and the bulk PBS [24]. Thereafter, in the next 7 days, the amount of GS released remained approximately stable at about 0.1 mg. In this second step, the kinetics of GS release is slower and remains under the control of a diffusion process from the deeper parts of ONTs. The mechanism of this extended GS release is due to the diffusive transport through the ordered ONTs in an insoluble matrix [24]. It is a surface-dependent process in which positively charged aminoglycoside with amino groups as prominent chemical groups of GS (Figure 9) undergo electrostatic interactions with the negatively charged ONT surface. Such kinetics of antibiotic release with a high local concentration of GS during the initial 6 h after the implantation guarantees the inhibition of the inflammatory processes in the surrounding tissues that occur immediately after implantation as a reaction to the appearance of a foreign body in the body. During this time, tissue-forming substances and growth factors are released into the body, but also the dynamic development of bacteria takes place.

**Figure 15:**
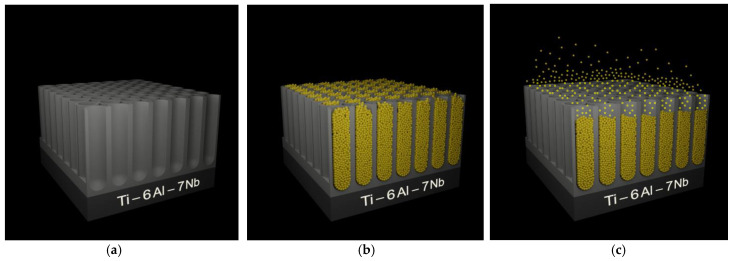
Scheme of oxide nanotubes (ONTs) on the Ti6–Al–7Nb alloy as a carrier of gentamicin sulfate (GS): (**a**) ONT layer after anodizing; (**b**) ONT layer loaded with GS; (**c**) release of GS molecules from ONTs immersed in phosphate-buffered solution. GS molecules are marked as yellow spheres.

Figure 15 shows a scheme of ONTs formed on the Ti6–Al–7Nb implant alloy under the proposed anodizing conditions for applications in targeted drug delivery systems. The obtained ONT layers can be proposed as drug carriers for local antibiotic therapy with extended drug release. The most common route of drug administration is the oral route. However, drug substances administered orally have a slower absorption. In an emergency, this way of administering the drug may not be effective. Orally administered drugs can disintegrate in the stomach and intestines, and in addition, the physiological environment of the human body causes drugs to lose stability and solubility in the gastrointestinal tract. Implants are often administered to elderly people who often suffer from other ailments. Orally administered medicinal substances irritate the stomach and burden the liver. In the case of oral administration of a drug substance, a preparation has to go a long way before it is absorbed into the epithelium to which it is to be administered [47,48]. The administration of a drug in controlled drug release systems enables an effective therapeutic dose to be obtained. The administration of the drug in such a system does not burden the organs additionally and ensures a constant, stable level of the drug. Compared to the oral administration of the drug, this system avoids a toxic dose of the drug.

The proposed Ti–6Al–7Nb/ONTs system with drug-eluting ability can not only inhibit infections and participate in bone healing, including in metastatic bone cancer, but also improve the osseointegration process due to the optimal porous structure.

It is worth adding that the drug-loading capacity of the formed ONTs can be fine-tuned to specific clinical needs, ensuring a sufficiently high local concentration of the drug to suppress bacterial infection. ONTs’ morphological parameters can be easily tailored by anodizing in 1 M EG + 0.2 M NH_4_ electrolyte. The obtained ONTs are also a universal carrier for various types of medicinal substances, substances with bone-forming abilities or other effects.

## 4. Conclusions

Based on the obtained results, it was found that the anodic oxidation of the vanadium-free Ti–6Al–7Nb alloy in 1 M EG + 0.2 M NH_4_F electrolyte in the voltage range of 15–100 V for 15–60 min is an effective method of producing third-generation ONTs.

The morphological parameters of the obtained single-wall ONTs are a function of the type and concentration of electrolyte, voltage, and anodizing time. By increasing the anodizing voltage, ONTs with larger internal diameters ranging from 45(5) to 155(25) nm and external diameters from 69(8) to 285(36) nm can be obtained. Extending the anodizing time while the voltage is not too high results in the formation of longer ONTs in the range of 0.281(1)–10.85(25) µm. The most optimal conditions for obtaining single-walled ONTs with smooth nanotube walls and the largest specific surface area are anodic oxidation using a voltage of 50 V for 60 min. Under such anodizing conditions, the longest ONTs with an inner diameter of 94(16) nm, an outer diameter of 144(17) nm, and a specific surface area of 600 cm^2^ cm^−2^ can be formed.

The characteristics of the physicochemical properties of the obtained amorphous ONT layers on the surface of the two-phase (α + β) Ti–6Al–7Nb alloy showed that they are mainly composed of metal oxides that are part of the chemical composition of the alloy, such as TiO_2_, Al_2_O_3_, and Nb_2_O_5_.

The functionalization of the surface of the biomedical Ti–6Al–7Nb alloy by producing self-assembling third-generation ONTs by anodic oxidation under the proposed conditions allows one to shape the morphology, microstructure, and thickness of the passive layer for applications in targeted drug delivery systems. The obtained ONTs revealed drug-eluting ability in the study of the model antibiotic loading (gentamicin sulfate) and its extended-release from the ONTs in phosphate-buffered solution. The proposed method of applying antibiotics with the locally deliver drugs allows one to skip oral supplementation and inhibit inflammation after implantation.

## Figures and Tables

**Table 1:** Morphological parameters of the ONTs formed on the Ti–6Al–7Nb alloy in 1 M EG + 0.2 M NH_4_F electrolyte.

Sample Number	*E*	*t*	*D*_i_	*D*_0_	*L*	*A*_s_
	(V)	(min)	(nm)	(nm)	(μm)	(cm^2^ cm^−2^)
1	15	60	45(5)	69(8)	2.02(32)	268
2	30	60	55(10)	89(9)	7.50(48)	327
3	50	60	94(16)	144(17)	10.85(25)	600
4	70	60	105(21)	146(19)	4.27(20)	333
5	100	60	155(25)	285(36)	2.40(24)	63
6	30	45	92(13)	135(15)	0.75(1)	106
7	50	30	115(14)	151(18)	0.28(1)	21
8	70	20	135(29)	209(40)	0.50(4)	18

## Data Availability

Not applicable.
